# Retinal Thickness Change Following Cataract Surgery in Eyes with Epiretinal Membranes

**DOI:** 10.3390/jcm13226781

**Published:** 2024-11-11

**Authors:** Barbara Wienerroither, Georgios Mylonas, Clemens Bernhart, Franz Prager, Guenal Kahraman, Michael Amon

**Affiliations:** 1 Department of Ophthalmology, Academic Teaching Hospital of Saint John of God, Johannes von Gott Platz 1, 1020 Vienna, Austria; babsi.wetzel@gmail.com (B.W.); michael.amon@bbwien.at (M.A.); franz_prager@yahoo.de (F.P.); guenal.kahraman@gmail.com (G.K.); clemensbernhart@hotmail.com (C.B.); 2 Sigmund Freud University Vienna, Campus Prater, Freudplatz 1, 1020 Vienna, Austria; 3 Department of Ophthalmology, Medical University of Vienna, Währinger Gürtel 18-20, 1090 Vienna, Austria

**Keywords:** epiretinal membrane, cataract surgery, retinal thickness, postoperative cystoid macular edema, ocular complications

## Abstract

**Purpose:** To investigate intraretinal changes and central millimeter thickness (CMMT) after successful uncomplicated cataract surgery in patients with asymptomatic epiretinal membrane (ERM). **Design:** prospective, single-center, interventional case series. **Methods:** Thirty eyes from 26 patients with cataract and ERM (ERM group) and fifteen consecutive eyes with senile cataract with a healthy macula (control group) were included. Best-corrected visual acuity (BCVA) and optical coherence tomography (OCT) as well as biomicroscopy were performed at baseline, one week, one month and three months after cataract surgery. OCT raster scans were further analyzed regarding CMMT and structural changes in the retinal morphology. **Results:** The baseline mean visual acuity improved significantly three months after cataract surgery in both groups (*p* < 0.05). CMMT in the ERM group was 342 (±53 μm) at baseline and increased significantly to 356 (±57) μm after three months (*p* < 0.05). One patient in the ERM group developed temporary cystoid macular edema one week after cataract surgery that resolved under topical treatment within three months. CMMT in the control group increased significantly three months after cataract surgery as well but no structural changes in the retinal morphology were observed in this group. **Conclusions:** This study provides evidence that cataract surgery in eyes with asymptomatic pre-existing ERM can be considered as safe and effective, resulting in good visual acuity outcomes. However, an increase in CMMT and temporary mild changes in retinal morphology may occur.

## 1. Introduction

Cataract is one of the leading global causes of blindness and cataract surgery is one of the most commonly performed operations worldwide [1]. The outcome of cataract surgery is based on the technical quality of the procedure and influenced by the presence of other ophthalmic diseases [2].

Optical coherence tomography is nowadays used in daily clinical practice and has become a helpful tool for the detection of even subtle macular changes also in the presence of opacification of the lens before cataract surgery [2]. Using recent high-resolution OCT technology, one can easily detect and analyze the early stages of epiretinal membranes (ERMs) and associated vitreomacular interface findings, such as macular cysts, paravascular cysts, lamellar macular holes, vitreomacular traction and full-thickness macular holes [3]. Clinicians and surgeons have to evaluate the need for treatment of these, often incidentally observed, macular findings and determine the best time for vitreoretinal surgery in patients with early stages of asymptomatic ERM.

The development of idiopathic ERM is known to be a result of a complex biochemical interaction between the retina and the vitreous fluid. ERMs typically occur after posterior vitreous detachment (PVD), due to a proliferation of retinal glial, fibroblasts and RPE cells on the anterior retinal surface. PVD potentially leads to microbreaks in the internal limiting membrane (ILM), allowing the migration of these cells through the retina [4,5].

Secondary ERMs may occur after surgery, inflammation, trauma or tumor of the posterior segment of the eye. However, it is still controversial whether the conditions in the anterior segment of the eye contribute to the formation of ERMs [6]. The contractile properties of ERMs, combined with their effect on the underlying retina, can result in considerable visual impairment and metamorphopsia.

Due to improved surgical techniques, cataract surgery has become increasingly safe [1]. However, the most frequent postoperative complication leading to visual impairment remains pseudophakic macular edema (PME)/Irvine–Gass Syndrome, which was first described by in 1953 by Irvine [7] and is caused by central retinal thickening due to serous exudation from incompetent intraretinal capillaries into the outer plexiform layer of Henle [8,9]. Studies reported an incidence of PME that varies from 3% up to 60%, depending on the diagnostic method (clinical examination, OCT or angiography) and the surgical method used. The incidence of PME after intracapsular cataract extraction is significantly higher than after extracapsular cataract extraction and the detection of PME is much easier using OCT and angiography, resulting in higher incidences compared to biomicroscopy [10,11]. Recent reported incidences of PME were observed to be between 0.2% and 2.35% after uneventful phacoemulsification surgery [12,13]. Several studies have explored the potential risk factors associated with macular edema following cataract extraction [14,15,16,17,18,19]. PME usually manifests 1 to 3 months after surgery, and resolves spontaneously (up to 80%) in most cases within 3 to 12 months postoperatively with full restoration of visual function [20].

The increasing utilization of OCT has significantly enhanced the ability to detect various retinal pathologies, including asymptomatic ERMs. Consequently, more clinicians are recognizing ERMs in patients who do not exhibit typical symptoms, which has led to greater awareness of this condition. This rise in detection prompts important clinical questions regarding the management of these patients, particularly concerning the timing and suitability of surgical interventions, such as cataract surgery, for those with asymptomatic ERMs.

The aim of this study was to investigate the structural morphology changes, CMMT and BCVA outcome after successful uncomplicated cataract surgery in patients with asymptomatic pre-existing ERM.

## 2. Patients and Methods

This study was designed as a prospective interventional case series. Following a standardized ophthalmologic examination, including Early Treatment of Diabetic Retinopathy Study (ETDRS), visual acuity, dilated pupil ophthalmoscopy, Amsler test and OCT imaging, 30 eyes of 26 consecutive patients meeting the inclusion criteria were entered in the clinical study.

For inclusion, patients had to have a visually compromising cataract and asymptomatic (without metamorphopsia) ERM with no structural changes such a cysts or photoreceptor distribution in OCT. Exclusion criteria were visual impairment or metamorphopsia related to ERM and other retinal pathologies like a history of branch retinal vein occlusion, central vein occlusion, wet or dry macular degeneration, presence of diabetic retinopathy in patients with diabetes mellitus, uveitis, or other inflammatory eye disease. Fifteen eyes without any retinal disease or previous ocular surgery with senile cataract were included as control group (Table 1).

Best-corrected visual acuity (BCVA), biomicroscopy and OCT were performed at baseline and one week, one month and three months after cataract surgery. The mean foveal thickness at the central 1-mm macular zone and the central 3-mm macular zone was assessed in OCT.

All examinations were conducted at the Department of Ophthalmology, Hospital of St. John of God, Vienna, Austria. The study followed the tenets of the Declaration of Helsinki and was approved by the local ethics committee. Before inclusion, all patients signed an informed consent form after a detailed discussion explaining the potential risks and benefits of the examination procedures. The study was registered in ClinicalTrials.gov (NCT03404323).

## 3. Surgical Technique

All surgeries were performed by two experienced surgeons (M.A., K.N.), using a standardized, small incision phacoemulsification technique and topical anesthesia. First, a 2.75 mm clear corneal incision was performed at the 12 o’clock position in all cases. Sodium hyaluronate 1% (Healon) was used as ophthalmic viscosurgical device and balanced saline solution for hydrodissection. Further, a well-centered continuous curvilinear capsulorhexis with a diameter of approximately 5 mm was created. After hydrodissection, endocapsular phacoemulsification of the nucleus and aspiration of the residual cortex were carried out. The capsular bag was filled with viscoelastic and the IOL was implanted in the bag using an injector system. Viscoelastic substance was then thoroughly evacuated by irrigation and aspiration.

All surgeries included in the study were uneventful; surgical complications like posterior capsule rupture, vitreous loss and prolapse through the wound or iris trauma were exclusion criteria.

Postoperative treatment consisted of topical bromfenac (Yellox) twice a day for 4 weeks. At month 1, patients with macular edema received additional topical Prednisolone acetate eye drops six times daily till month 3 follow-up time. In the case of persistent edema at the month-3 follow-up, an injection of intravitreal triamcinolone (4 mg) was applied.

## 4. Statistics

All data were entered into a Microsoft Excel spreadsheet. To detect differences in 1 mm thickness (CMMT) and 3 mm ETDRS ring (nasal, temporal, superior and inferior) values at each time of examination, we used a paired *t* test. A *p* value of less than 0.05 was considered statistically significant. Statistical analysis was performed by SPSS 17.0. software (SPSS Inc., Chicago, IL, USA).

## 5. Results

The age range of the study patients was 75 ± 6 years (mean ± standard deviation [SD]) and 74 ± 4 years in the control group. The ERM group consisted of 30 eyes from 26 patients, 13 male and 13 female, 14 eyes were right eyes. In the control group, 9 right eyes and 6 left eyes of 5 male and 10 female patients were included. Twenty-nine eyes in the ERM and 15 eyes in the control group completed the examinations and remained in the analysis until month 3; one patient was not available for follow-up in the ERM group.

The baseline mean ± SD BCVA in the ERM group was 70 ± 7 ETDRS letters, which improved significantly to 78 ± 6 ETDRS letters 1 week after cataract surgery (*p* < 0.001) and stayed relatively stable at the 1 and 3 months follow-up visits; 78 ± 6 and 79 ± 7 ETDRS letters for 1 and 3 months, respectively, (difference baseline/1 and 3 months: all *p* < 0.001).

The mean (±SD) CMMT was 342 (±53) μm at baseline and increased significantly to 352 (±57) μm at the one week visit (*p* < 0.05). After one month, the highest CMMT values were observed with 359 (±56) μm and they then stayed relatively stable until the 3-month follow-up visit; 356 (±57) μm (CMMT difference baseline/1 and 3 months: *p* < 0.001).

Regarding central 3 mm thickness, similar results were noted in the ERM group. Baseline 3 mm thickness was 352 (±31) μm and increased to 357 (±32) μm one week after surgery. At month 1, a further increase to 361 (±32) μm was observed, and then the thickness values stayed stable until month 3 (361 (±32) μm (difference baseline/1 week, 1 and 3 months: all *p* < 0.001).

In the control group, the baseline mean ± SD BCVA was 69 ± 7 ETDRS letters and improved significantly to 81 ± 4 ETDRS letters at 1 week after surgery (*p* < 0.001). One month after surgery, mean ± SD BCVA changed to 83 ± 3 ETDRS letters and after 3 months, mean ± SD BCVA reached its maximum with 84 ± 3 ETDRS letters, representing significant improvement compared to baseline (*p* < 0.001).

The baseline mean (±SD) CMMT in the control group was 257 (±22) μm, decreasing to 254 (±21) μm one week after surgery. An increase in CMMT compared to baseline was noticed at month 1, where mean (±SD) CMMT values were 260 (±25) μm, and reached its maximum of 261 (±23) μm at the 3-month follow-up visit. Compared to baseline, the rise in CMMT in the control group was statistically significant only 3 months after surgery (*p* < 0.05).

The baseline mean (±SD) central 3 mm thickness in the control group was 307 (±16) μm and did not change one week after surgery 307 (±16) μm. At month 1, central 3 mm thickness increased significantly to 312 (±18) μm and then stayed unchanged at the 3 months follow-up visit (difference baseline/1 and 3 months: all *p* < 0.05).

**Figure 1:**
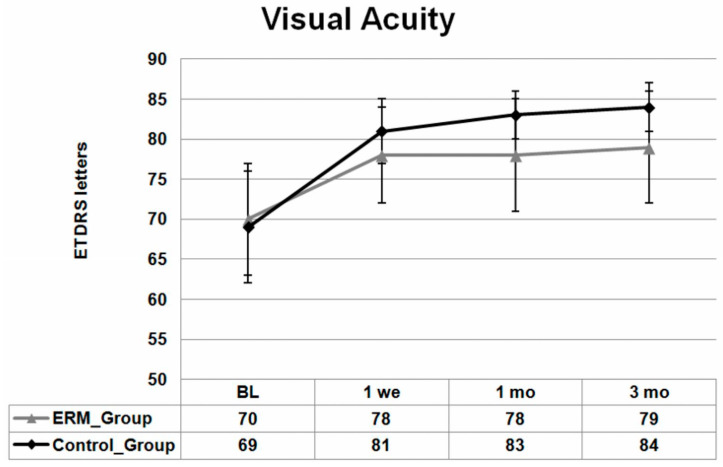
Mean ± standard deviation (SD) changes from baseline best-corrected visual acuity (BCVA) of both groups over 3 months in ETDRS letters. Mean BCVA improved significantly compared with the baseline in both groups in all visits during the follow-up period (all *p* ≤ 0.05).

**Figure 2:**
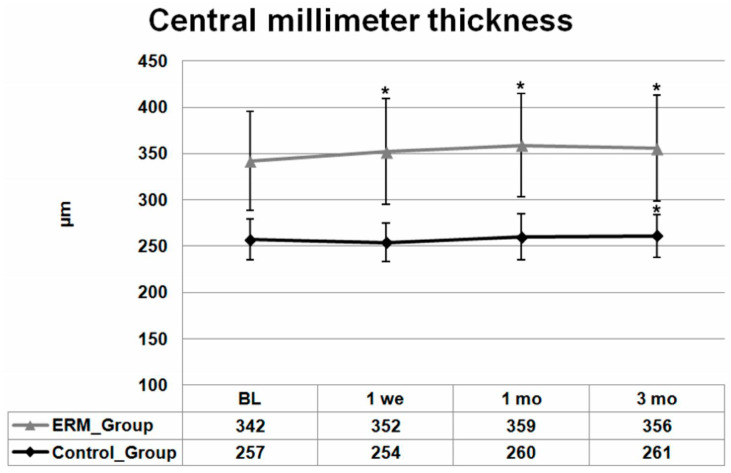
Mean ± standard deviation (SD) changes from baseline central millimeter thickness (CMMT) of both groups over 3 months. Asterisks indicate a significant difference between baseline and each follow-up visit examination (*p* ≤ 0.05).

**Figure 3:**
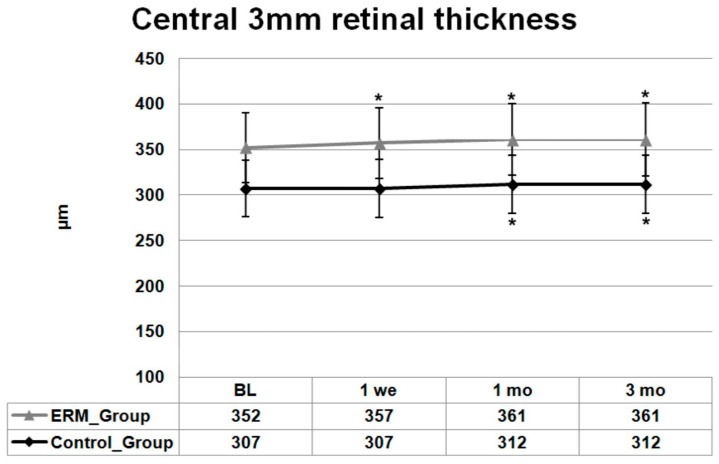
Mean ± standard deviation (SD) changes from baseline central 3 mm retinal thickness values of both groups over 3 months. Asterisks indicate a significant difference between baseline and each follow-up visit examination (*p* ≤ 0.05).

BCVA, CMMT and 3 mm thickness measurement changes with SD over the follow-up time are presented in Figure 1, Figure 2 and Figure 3, respectively.

## 6. Changes in Retinal Structural Morphology by Cirrus HD-OCT

Although retinal thickness increased significantly in all patients in the ERM group, in 28 of 29 eyes, no remarkable changes in retinal morphology (such as cysts or intraretinal or subretinal fluid) were observed. However, one patient in the ERM group developed cystoid macular edema one week after cataract surgery.

This patient presented with visual acuity impairment and metamorphopsia corresponding to the retinal thickening and intraretinal cyst formation in the OCT examination.

At the three-month visit, cystoid macular edema had completely resolved under topical treatment with prednisolone acetate eye drops (Prednifluid) and bromfenac eye drops (Yellox), resulting in the recovery of visual acuity and metamorphopsia as well.

In the healthy control group, no definite structural changes in the retinal morphology were observed despite a significant increase in CMMT at month 3.

## 7. Discussion

This prospective study investigated the structural retinal integrity regarding the functional and morphologic parameters in eyes with pre-existing asymptomatic ERM after successful cataract surgery without any surgical complications using HD-OCT imaging.

It is thought that extracapsular cataract extractions with larger incisions have more influence on vitreous changing processes, and therefore may cause accelerated progression of ERM [6,21,22]. Although there is still controversy, it can be assumed that advances in cataract surgery, such as small-incision phacoemulsification have contributed to the decrease in the progression of ERM after surgery. In fact, even uneventful small-incision cataract surgery has the potential of inducing inflammatory and mechanical changes in the vitreoretinal structures causing macular thickening. Various soluble mediators seem to be responsible for multiple sites of leakage resulting in damage and break-down of the blood–retinal barrier [23,24]. All of our study patients showed a significant increase in central macular millimeter thickness three months after phacoemulsification surgery without any negative effect on the visual function. Similar results have already been reported by two other studies which analyzed central macular thickness changes after cataract surgery in several different groups of patients [25,26]. It was demonstrated that macular thickness values in patients with ERM remained significantly higher even 6 months after surgery without preventing optimal recovery of visual function [26].

Another study showed that there is no linkage between acceleration of progression of ERM and uneventful phacoemulsification surgery, by comparing eyes with ERM that underwent surgery with eyes that did not [6]. A significant increase in foveal thickness and macular volume was reported in all eyes as well after a 12-month follow-up.

Various studies investigated the occurrence of changes in retinal integrity like pseudophakic cystoid macular edema (PCME) after cataract extraction and highlighted several predisposing potential risk factors including complicated phacoemulsification surgery (e.g., rupture of the posterior capsule, loss or prolapse of vitreous through wounds, iris trauma), diabetic retinopathy, uveitis and previous vitrectomy with peeling of the internal limiting membrane [27,28,29,30]. In our ERM study group, one patient developed temporary cystoid macular edema after surgery, which resolved completely under topical treatment with eye drops after three months. According to previously published data, preexisting ERM is also known to carry a higher risk of developing PCME after cataract extraction [31,32]. Nevertheless, in these studies, no standard OCT examination was performed in every patient before surgery; ERMs were only diagnosed clinically. Therefore, the higher risk of PCME might be associated with the existence of preoperative clinically obvious ERM in these study groups. Our results in the patient group with asymptomatic early stages of ERM showed that uneventful phacoemulsification does not lead to a significantly higher risk of developing PCME after a three-month follow-up period.

In the healthy control group, no cases of PCME were observed. However, all patients in the control group showed a significant increase in central macular thickness values three months after surgery as well. This is in keeping with previously published data since variations in retinal thickness, comparing values before and after cataract surgery in eyes with a healthy macular, have been described in detail by a number of studies, always confirming that no influence on visual function could be noticed so far [2,25,26,33,34].

One of the main limitations in our study was the fact that we were not able to use an integrated automatic eye-tracking system while performing the raster scans in our OCT measurements and instead of that, an experienced reader centered the scans manually. However, in order to guarantee the reliability of our measurements, we evaluated the central 1 mm macular zone and additionally the central 3 mm macular zone as well. No significant differences were noted in the ERM group between these two different measurements; only in the control group, the measurements of the central 3 mm macular zone reached statistically significant increased values slightly earlier compared to baseline (1 month after surgery in relation to the measurements of the central 1 mm thickness which showed significant increase only at month 3). Another limitation of our study is the relatively small sample size and the short follow-up period of three months, which could yield different conclusions with a larger population and an extended duration. However, since this was a pilot study, further research is necessary to validate our findings.

In conclusion, this prospective study demonstrates that small incision cataract surgery is a safe option for patients with cataracts and early-stage ERM who do not exhibit clinical symptoms such as metamorphopsia. While our findings revealed a significant increase in macular thickness, this did not impede the optimal recovery of visual function in our study cohort. However, it is important to recognize that temporary mild changes in retinal morphology may occur, and both patients and healthcare providers should remain mindful of this potential risk. We recommend utilizing OCT to monitor for PCME and to assess visual acuity in these patients, as additional treatment may be warranted if improvements are not observed. Furthermore, it is essential to conduct additional observations and investigations with a larger sample size and longer follow-up period to confirm our findings.

## Figures and Tables

**Table 1:** Inclusion and exclusion criteria of our study population.

Inclusion Criteria:	Exclusion Criteria:
Visually compromising cataract. Asymptomatic epiretinal membrane (ERM) without metamorphopsia. No structural changes in the retina on OCT, such as cysts or photoreceptor disruption.	Visual impairment or metamorphopsia related to ERM. History of branch retinal vein occlusion. History of central retinal vein occlusion. Wet or dry age-related macular degeneration. Diabetic mellitus Uveitis or other inflammatory eye diseases.
Control Group Inclusion Criteria	
No retinal disease. No history of previous ocular surgery. Presence of senile cataract.	

## Data Availability

The original contributions presented in this study are included in the article. Further inquiries can be directed to the corresponding author(s).
